# Overcoming Platinum and PARP-Inhibitor Resistance in Ovarian Cancer

**DOI:** 10.3390/cancers12061607

**Published:** 2020-06-17

**Authors:** Michelle McMullen, Katherine Karakasis, Ainhoa Madariaga, Amit M. Oza

**Affiliations:** Division of Medical Oncology & Hematology, Bras Family Drug Development Program, Princess Margaret Cancer Centre, University Health Network, Toronto, ON M5G 2M9, Canada; michelle.mcmullen@uhn.ca (M.M.); Katherine.Karakasis@uhn.ca (K.K.); ainhoa.madariaga@uhn.ca (A.M.)

**Keywords:** resistance mechanisms, platinum, PARP, ovarian cancer, homologous recombination

## Abstract

Platinum chemotherapy remains the cornerstone of treatment for epithelial ovarian cancer (OC) and Poly (ADP-ribose) polymerase inhibitors (PARPi) now have an established role as maintenance therapy. The mechanisms of action of these agents is, in many ways, complementary, and crucially reliant on the intracellular DNA Damage Repair (DDR) response. Here, we review mechanisms of primary and acquired resistance to treatment with platinum and PARPi, examining the interplay between both classes of agents. A key resistance mechanism appears to be the restoration of the Homologous Recombination (HR) repair pathway, through *BRCA* reversion mutations and epigenetic upregulation of *BRCA1.* Alterations in non-homologous end-joint (NHEJ) repair, replication fork protection, upregulation of cellular drug efflux pumps, reduction in PARP1 activity and alterations to the tumour microenvironment have also been described. These resistance mechanisms reveal molecular vulnerabilities, which may be targeted to re-sensitise OC to platinum or PARPi treatment. Promising therapeutic strategies include ATR inhibition, epigenetic re-sensitisation through DNMT inhibition, cell cycle checkpoint inhibition, combination with anti-angiogenic therapy, BET inhibition and G-quadruplex stabilisation. Translational studies to elucidate mechanisms of treatment resistance should be incorporated into future clinical trials, as understanding these biologic mechanisms is crucial to developing new and effective therapeutic approaches in advanced OC.

## 1. Introduction 

Epithelial Ovarian Cancer (EOC) is the seventh most common cancer in women and the leading cause of gynecologic cancer death worldwide [1]. For the last three decades, platinum-based chemotherapy has been the cornerstone of systemic treatment for EOC [2,3,4]. Standard front-line treatment for advanced EOC consists of cytoreductive surgery, with the goal of no residual disease (R0), and platinum-based chemotherapy [5]. At the time of relapse, if the time interval since the last dose of platinum chemotherapy (Treatment Free Interval-Platinum; TFIp) is more than six months, the disease is considered platinum sensitive, and standard treatment consists of re-challenge with platinum [5]. The majority of high-grade serous ovarian cancers (HGSC) are initially platinum sensitive. However, even with optimal treatment, HGSC will typically follow a frequent relapse-response pattern, before eventually becoming platinum resistant [5]. Maintenance treatment has emerged as an important strategy to prolong the period between treatment responses and disease relapse [6]. The Poly-ADP Ribose Polymerase inhibitors (PARPi) have demonstrated impressive activity in the first [7,8,9] and second-line [10,11] maintenance settings. This benefit does is not limited to patients with germline or somatic *BRCA* mutation (*BRCAm*), or patients with other forms of Homologous Recombination Deficiency (HRD) [12]. Unfortunately, as with platinum chemotherapy, many if not most patients will eventually acquire resistance to PARPi treatment. Outcomes in platinum resistant EOC are extremely poor, with median survival of only 12 months [5]. There is consequently an urgent need to elucidate the mechanisms of platinum and PARPi resistance in EOC to improve patient stratification for therapeutic strategies that target molecular vulnerabilities to overcome treatment resistance. In both cases, mechanisms appear to be complex and inter-related. Resistance to platinum chemotherapy is strongly predictive of resistance to PARPi treatment. Cross resistance between two different therapeutic classes points to overlap in biologic mechanisms of susceptibility and resistance [13]. This article will review the literature regarding mechanisms of treatment resistance to platinum and PARPi in EOC and highlight planned strategies and clinical trials that may effectively re-sensitise tumours to these agents. 

## 2. Alterations in DNA Damage Repair Can Drive Treatment Resistance

The DNA Damage Repair (DDR) response is designed to detect DNA damage, and initiate cell repair in order to maintain genomic integrity within the cell [14]. It consists of a complex network of inter-related signalling pathways. The “master sensors” (ATM, ATR, and DNA-PKs) are large serine/threonine kinases, which sense DNA damage and initiate repair signalling cascades by phosphorylating key proteins such as BRCA1, CHK1, CHK2, p53 and RAD17 [15]. The activation of signalling-transduction pathways promotes activation of DNA-damage-dependent cell checkpoints that slow or halt cell cycle progression, allowing more time for DNA repair [14]. To date, six main DDR pathways have been described; Homologous Recombination (HR), Non-Homologous End Joining (NHEJ), Base Excision Repair (BER), Nucleotide Excision Repair (NER), and Fanconi Anaemia (FA) pathway, and Mismatch Repair (MMR) [14]. 

Alterations in these DDR pathways have dual significance in OC. In oncogenesis, alterations in DDR leading to genomic instability are a hallmark of cancer development [16]. Germline mutations in HR pathway genes (*BRCA 1/2*) result in a 14–44% cumulative lifetime risk of EOC [17], with the contribution of other genes becoming increasingly apparent [18]. However, these same alterations in DDR pathways present molecular vulnerabilities, which are targetable by anti-cancer therapy (Table 1). Both platinum chemotherapy and PARPi are genotoxic agents, which exploit defects in DDR pathways to affect cancer cell death [19]. Alterations in DDR (including upregulation or downregulation of key effectors) can drive sensitivity or resistance to these agents (Table 2) [20]. 

### 2.1. HRD Conveys Sensitivity to Platinum and PARPi

Platinum chemotherapy exerts its cytotoxic effect by binding with DNA to form mono-adducts. These mono-adducts then evolve through second covalent binding to a DNA crosslink (commonly intra-strand, but also inter-strand), resulting in structural change to the DNA double helix. Platinum treatment leads to an accumulation of DNA double-strand breaks (DSBs) [21]. Tumours with HRD demonstrate a higher degree of chromosomal instability and are fatally unable to repair DSBs, enhancing sensitivity to platinum agents [21]. 

The PARP family of proteins (especially PARP1) are essential for sensing single-strand DNA breaks (SSBs), initiating repair via predominantly the BER pathway [22]. PARPi are able to block the activity of PARP1 for the repair of SSBs, and trap PARP1 on damaged DNA, posing an insurmountable block to the replisome [22]. The accumulation of SSBs leads to the development of fatal DSBs. Cells require a functional HR repair pathway to resolve these replisome blocks and resume cell-cycle progression, and to repair DSBs [23]. Consequently, PARPi exploit the concept of synthetic lethality in HR-deficient tumours, in order to effect cancer cell death [23]. 

Some form of HRD is likely to be present at baseline in more than 50% of HGSC potentially underlying the initial sensitivity of this type of tumour to DNA damaging drugs, such as platinum chemotherapy [24]. HR functions to repair DSBs or stalled replication forks during the S and G2 phases of the cell cycle [25]. The pathway is initiated by BRCA1, and uses a sister chromatid as a template for DNA repair, via recruitment of the MRN complex, BRCA2 and RAD51 [19]. Deregulation of HR repair pathway results in chromosomal instability—a phenotype referred to as ‘BRCAness’—which increases the sensitivity of the tumour to DNA damaging drugs [23]. HRD may be the most clinically relevant molecular stratification in OC, and it is an important predictive biomarker for response to both platinum chemotherapy and PARPi [26]. However, the definitive classification of HRD has proven to be elusive. A number of HR assays are undergoing validation (i.e., BROCA, Myriad, Foundation Medicine, HRD detect) [27]. These assays are necessarily limited in their capacity to consider clinically relevant changes such as epigenetic alterations, or to characterise for spatial or temporal tumour heterogeneity within an individual patient. The complexity of defining HRD is increased by the fact that a small subset of patients with HRD never demonstrate a response to platinum and PARPi treatment, whilst some patients without HRD respond well to these same treatments. Any discussion of the key role of HRD in platinum and PARPi mechanisms of action must also consider that multiple and overlapping DDR pathways are likely involved in determining the response, and HRD alone is probably insufficient to predict response to treatment. 

### 2.2. Reactivation of HR Is a Mechanism of Acquired Resistance 

Restoration of the function HR pathway in HRD tumours is a key mechanism of acquired platinum and PARPi resistance, which has been demonstrated in vitro and in vivo (Table 3). Restoration of HR may be achieved by secondary mutations, which restore the open reading frame of the *BRCA* gene, functionally restoring protein activity (BRCA reversion) [28,29,30]. *BRCA* reversion mutations have been described in both progression biopsies and cell-free DNA (cfDNA), in patients with acquired resistance to PARPi [31,32,33,34,35]. Some cfDNA studies have been able to demonstrate a polyclonality of multiple reversion mutations within a single patient, illustrating that treatment exerts profound selective pressure to restore BRCA1/2 protein activity and overcome PARPi sensitivity [31]. Reversion mutations in HR genes have also been described as acquired resistance mechanisms to platinum chemotherapy [33]. Reversion mutations in *BRCA* and other HR genes have also been observed in PARPi resistant tumours, including *RAD51C/RAD51D* reversion mutation in patients with acquired resistance to rucaparib [36]. Other mechanisms that restore BRCA function have been reported in platinum and PARPi resistant cancers, including the loss of *BRCA1* promoter methylation [28,37]. Additionally, functional restoration of mutant BRCA1 protein may be achieved through interaction with HSP90, which can promote RAD51 loading onto DNA following DNA damage [38]. Interestingly, in the absence of *BRCA* reversion mutation, an increase in BRCA1 protein expression has been observed in patients with BRCA1 C-terminal domain mutations [39], suggesting alternative mechanisms to *BRCA* reversion mutation for reactivating HR. The OC genome appears to display a significant degree of adaptability in response to selective pressure of treatment, with reactivation of HR occurring by multiple different mechanisms, to enable cancer cell survival in the presence of Platinum and PARPi treatments. 

### 2.3. Non-Homologous End Joining (NHEJ) 

**Figure 1:**
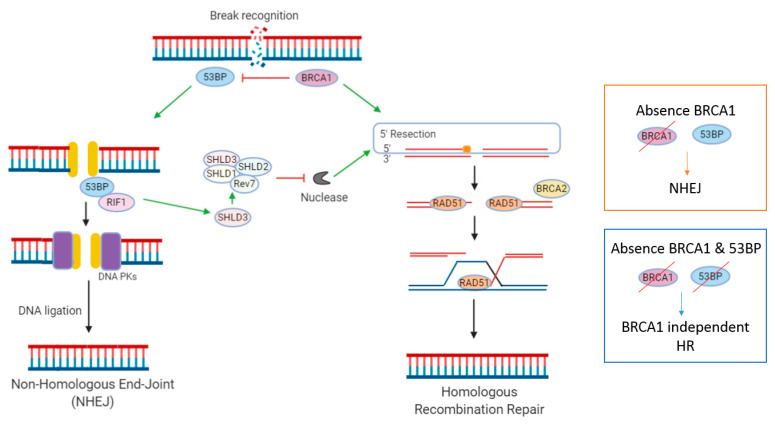
DNA Double Strand Break repair by Homologous Recombination or Non-Homologous End-Joining pathway. Figure: In the absence of functional BRCA1, DNA Double strand breaks (DSBs) may be repaired by the Non-Homologous End Joining (NHEJ) pathway. Loss of 53BP1 is an acquired resistance mechanism in BRCA1 mutant tumours. 53Bp1 normally promotes DSB repair by NHEJ, by forming a 53Bp1-RIF1 complex that protects DSB ends from endonuclease processing. Loss of 53BP1 prohibits a recruitment of Shieldin (SHLD) complex to DSB, allowing 5′ end resection, and reactivation of HR in a BRCA1 independent manner.

NHEJ is the second important DDR pathway involved in the repair of DSBs. In contrast to HR, which initiates repair during S and G2 phases of the cell cycle, NHEJ occurs throughout interphase [19]. Multiple regulatory mechanisms dictate whether DSBs are repaired via the NHEJ or HR pathway. A key mechanism appears to be the antagonism between HRR-promoting factor BRCA1 and NHEJ-promoting factor 53BP1 [40] (Figure 1). 53BP1 promotes DSB repair by NHEJ in G1 phase cells, by forming 53BP1-RIF1 complexes that protect DSB ends from exonuclease processing [40]. The loss of BRCA1 in HRD tumours can be overcome by concomitant loss of 53BP1, resulting in HR reactivation, rendering cells resistant to the synthetically lethal approach of PARPi (Figure 1) [40]. This re-wiring of DDR pathways via loss of 53BP1 has been described in numerous in vitro studies [41,42,43]. This was first reported by three landmark papers, in which it was observed from mouse models that HRD caused by *BRCA1m* could be reversed by concomitant loss of 53BP1 (TP53BP1), a protein involved in NHEJ repair [44,45,46]. Interestingly, loss of 53BP1 does not seem to restore HR function in *BRCA2m* cells, a finding which may be relevant when considering predictive biomarkers and patient stratification.

PARPi-resistance CRISPR/Cas9 screens have identified that the Shieldin (SHLD) complex is actively involved in inhibiting DNA resection [40,42]. Here, SHLD proteins (Rev7, SHLD1, SHLD2, SHLD3) are recruited to DSBs via SHLD3 in a 53BP1 and RIF1-dependent manner. SHLD then blocks nucleases at DSBs, thereby inhibiting 5′ end resection. Resection at 5′ends of DSBs is essential for initiation of repair by the HR pathway. When resection is lost, HR can be re-initiated in a BRCA-independent fashion, via recruitment of PALB2. CRISPR/Cas9 screens have also identified DYNLL1 and HELB as, respectively, 53BP1-dependent and 53BP1-independent, mediators of PARPi resistance [40]. 

### 2.4. Nucleotide Excision Repair 

The Nucleotide Excision Repair (NER) pathway is a highly conserved mechanism to remove “bulky lesions”, which distort the DNA double helix, including the intra-strand crosslinks formed by platinum adducts [19]. The NER pathway is altered in about 8% of EOC at baseline [47]. The NER pathway defect has been associated with improved Overall Survival (OS) and Progression Free Survival (PFS), suggesting that NER pathway inactivation could confer platinum sensitivity [47]. Interestingly, however, in vitro data demonstrates that NER alteration does not confer sensitivity to PARPi, suggesting that PARP and platinum sensitivity do not always occur in parallel [48]. ERCC1 and XPF are the two components of NER that are potentially most important for removal of platinum-DNA adducts [49]. ERCC1 complexed with XPF is involved in the 5′ cleavage of DNA strands that carry platinum adduct. This complex also plays a role in the HR pathway, repairing intra-strand crosslink. There is conflicting data regarding the validity of ERCC1 as a predictive biomarker of platinum resistance [47,49,50,51,52,53,54,55,56,57]. Polymorphisms in other NER components (i.e., XPA, XPB, XPF, XPD) have shown no correlation with platinum resistance [58]. 

### 2.5. Replication Fork Protection 

In a normal cell cycle, replication forks may stall in response to replication stress, prolonging S-phase arrest so that DNA repair can occur [59]. In addition to their role in HR, BRCA1 and BRCA2 bind to stalled replication forks, prohibiting the recruitment of MRE11 nuclease, protecting forks from excessive nuclease degradation and stabilising the replication fork [60]. 

Loss of replication fork protection elicits genomic instability, which initially promotes tumourigenesis. However, replication fork stabilisation by BRCA-independent mechanisms has been described as a mechanism of acquired treatment resistance, promoting cancer cell progression through the cell cycle even in the presence of DNA damage and replication stress. For example, overexpression of RAD51 has been reported in *BRCAm* cells, restoring RAD51 loading and replication fork protection [59,61,62,63]. SLFN11 is another regulator of S-phase, recruited to stressed replication forks, and able to open chromatin and block replication, prolonging S-phase arrest in the presence of DNA damage. Loss of SLFN11 has been described as a mechanism of acquired platinum resistance [64,65]. Pax2 transactivation domain-interacting protein (PTIP) and Chromodomain Helicase DNA Binding Protein 4 (CHD4) promote recruitment of MRE11 nuclease to replication forks [59]. Lower PTIP expression and CHD4, prohibiting MRE11-dependent onceolytic degradation of nascent DNA, have also been associated with replication fork stabilisation and platinum resistance [59]. 

The Fanconi Anaemia (FA) repair pathway is important for removing intra-strand DNA crosslinks, formed by platinum adducts [66]. Additionally, this pathway coordinates DNA replication, fine-tuning mitotic checkpoints to ensure error-free chromosomal segregation [66]. The FA pathway is important for replication fork stabilisation, and mutations in FA pathway genes may have similar effects to BRCA1 and BRCA2 mutation, in promoting progression of cancer cells through the cell cycle, even in a setting of DNA damage and replication stress [59]. 

### 2.6. Reduced Cellular Availability of Drugs 

Reduced cellular availability of chemotherapeutic drugs has been reported as a mechanism of acquired resistance to both platinum chemotherapy and PARPi in ovarian cancer. The copper transporters CTR1, CTR2, ATP7A, and ATP7B regulate intracellular concentration of platinum by mediating its uptake and efflux in cells [67,68,69]. Overexpression of ABCB1, the gene that encodes Multidrug Resistance protein 1 (MDR1), an ATP-binding cassette member involved in the cellular efflux of chemotherapeutic drugs, has also been reported as an acquired resistance mechanism to PARPi [70,71,72]. Upregulation of MDR1 has been described in an engineered PARPi-resistant human ovarian cancer cell line [72], as a result of chromosomal translocations involving the ABCB1 gene [73]. A Whole Genome Sequencing (WGS) study of ovarian cancer cells from matched primary and recurrent ascites, demonstrated upregulation of ABCB1 (through promoter fusion and translocation involving the 5′ region of the gene) in approximately 8% of recurrent HGSOC samples [28]. Most PARPi are MDR1 substrates [74], and this data suggests that prior paclitaxel chemotherapy may precondition tumours to be resistant to PARPi via upregulation of MDR1. The co-administration of MDR1 inhibitors with PARPi is a strategy that has not yet been explored in clinical trials [75], but future research may focus on targeting patients with *ABCB1* mutations involved in PARPi resistance. Alteration in intracellular proteins that are able to bind and sequester platinum (including metallothioneins and glutathione [76,77]), and altered expression of pro-survival or anti-survival proteins have also been described as resistance mechanisms which reduce cellular availability of drugs. Proteomic analysis of paired primary and recurrent OC cells from ascites has revealed that RELA and STAT5 proteins cooperate in inducing the anti-apoptotic Bcl-X promoter activity and synergistically enhance *Bcl-xL* expression in chemo-resistant ovarian cancer cells [78].

## 3. Immunosuppressive Tumour Microenvironment 

These genomic alterations in DNA damage response, must be considered in conjunction with the tumour microenvironment when attempting to elucidate a broad mechanistic understanding of resistance mechanisms in ovarian cancer. Detailed discussions of biomarkers of response to immunotherapy and anti-angiogenic therapy are beyond the scope of this article. However, broadly speaking, immunosuppressive changes within the tumour microenvironment have been associated with resistance to chemotherapy. The increased infiltration of immunosuppressive CD163+ macrophages [79,80], and increased infiltration of regulatory FOXP3+ T cells l [81], has been shown to favour tumour growth, in contrast to the presence of tumour infilitrating lymphocytes (TILs) which is positively correlated with survival [82]. PD1, PD-L1 expression and Tumour Mutational Burden have not demonstrated consistent validity as predictive biomarkers for immune checkpoint inhibition in ovarian cancer. Retinoic acid-inducible gene-I (RIG-I) overexpression is associated with poor-prognosis platinum resistant and refractory cancers [83]. Importantly, RIG-I overexpression was also associated with local immunosuppressive changes such as increased interferon production, and a distinct immune-regulatory signature involving immune checkpoint molecules (PD-L1/PD-1), the RNA editing enzyme ADAR1 and regulatory FOXP3+ T cells [83].

Stromal activation, including extensive stromal desmoplasia, has also been reported to be associated with acquired treatment resistance [28]. It appears to be a key phenotypic characteristic of the chemo-resistant “mesenchymal” molecular subgroup defined in The Cancer Genome Atlas (TCGA) network study [84]. 

## 4. Targeting Molecular Vulnerabilities to Overcome Treatment Resistance

### 4.1. Targeting ATR

Ataxia telangiectasia and Rad3-related (ATR) protein is a key kinase at the heart of the DDR, responsible for sensing replication stress and signalling to S and G2/M checkpoints to initiate repair [15]. ATR inhibitors may be able to reduce the rate of DNA repair in cells, thereby increasing DNA damage and causing cell death [85]. However, single agent ATR inhibition appears to be less effective than synergistic combinations, that increase the effect of synthetic lethality in tumours with DNA repair deficiencies [86]. Combined with PARPi, the simultaneous inhibition of two repair pathways during S-phase, with abrogation of the S/G2 cell cycle checkpoint, leads to accumulation of DSBs in actively replicating cancer cells and cell death in the M-phase [85,86]. The DUETTE study (NCT04239014), will assess the combination of Ceralasertib (AZD6738)—a potent, selective ATR inhibitor—in combination with Olaparib, as second maintenance therapy in patients who have platinum-sensitive OC, who have acquired resistance from prior PARPi treatment. Another ongoing clinical trial is combining the ATR inhibitor M6620 with the PARP inhibitor veliparib to evaluate if this combination can impair DNA repair and induce the ‘*BRCA*ness’ phenotype in solid tumours, including ovarian cancer, which may increase sensitivity to platinum chemotherapy (NCT02723864) [87].

### 4.2. Epigenetic Resensitisation 

It has been observed that the acquisition of a treatment resistant phenotype is associated with the accumulation of epigenetic changes. These include transcriptional silencing of tumour suppressor and DNA repair genes, including *BRCA1*, *TP53*, *PTEN* and *MLH1* [88,89]. It is proposed that epigenetic modulators may be able to re-sensitise tumours to platinum-chemotherapy. The DNA methyltransferase (DNMT) inhibitors have not been effective as a single agent treatment in platinum resistant OC. However, in combination they may be able to enhance sensitivity to platinum by altering epigenetic regulation of gene expression. Mixed results have been observed in clinical trials. Decitabine administered one week prior to carboplatin initially demonstrated no activity [90]. It was proposed that this may have been related to the dosing schedule. At low dose, administered continuously in combination with carboplatin, decitabine was shown to reduce DNA methylation of genes in cancer pathways and apoptosis [91,92]. Azacitadine in combination with carboplatin was investigated in a phase Ib/IIa trial, with an objective response rate (ORR) of 13.8% observed in a platinum resistant OC population [93]. In a randomised phase II study [94], assessing Guadecitabine in combination with carboplatin, the ORR and clinical benefit rate was 15% and 45%, respectively. Correlative analyses of this study, including transcriptomic analysis, demonstrated that inhibiting DNA methylation can sensitise OC cells to platinum drugs, in part by altering gene expression patterns related to DNA repair and immune activation, an approach warranting further investigation [95].

The DNA damage initiated by DNMT inhibitors is repaired by the BER pathway, of which PARP1 plays a central role. Additionally, it has been proposed that DNMT treatment induces a “BRCAness” phenotype [96,97]. In light of this, there may be a mechanistic rationale to investigate the combination of DNMT inhibitors with PARPi. This therapeutic approach is yet to be explored in clinical trials.

Histone Deacetylase (HDAC) inhibitors are another class of epigenetic modulator that have not shown significant anti-tumour activity as single agents in platinum resistant OC. A phase II study [98], assessing the combination of Belinostat with carboplatin (*n* = 29), was stopped early due to lack of activity. Another phase II study assessing Belinostat in combination with carboplatin (*n* = 35) and paclitaxel [99] demonstrated an ORR 43%. The discrepancy between anti-tumour activity observed between these studies may be related to heterogeneity in study populations, small sample size and potentially the independent cytotoxic effect of paclitaxel.

### 4.3. Cell Cycle Checkpoint Inhibitors 

The cell cycle checkpoint regulators, CHK1/2, halt cell division to allow DNA damage to be repaired prior to DNA replication [100,101]. Cell cycle checkpoint inhibition may be able to prevent progression of cancer cells through cell-cycle, halting replication and tumour growth. 

Prexasertib is a CHK1/2 inhibitor, which demonstrated an ORR of 29% in non-g*BRCA*m carriers in a phase II study in recurrent (predominantly platinum-resistant) HGSOC cohort (NCT02203513) [102]. The results from the germline *BRCAm* cohort of this study is awaited. 

CHK1/2 inhibitors are also being tested in combination with platinum chemotherapy (NCT02797977) and PARP inhibitors (NCT03057145), to assess possible synergy, and if combination treatment can amplify the effects of DNA damage and increase CHK1/2 inhibition-related apoptosis.

WEE-1 inhibitors act on WEE-1 kinase, a G2 cell-cycle checkpoint regulator, in order to abrogate G2 cell cycle arrest, and enhance cancer cell apoptosis in the setting of DNA damage [103]. A phase II proof-of-concept trial [104] assessed the combination of WEE-1 inhibitor, (AZD1775) and carboplatin in *TP53* mutated platinum resistant OC, demonstrating an ORR of 43%. Correlative transcriptomic analyses revealed that *BRCA1*, *MYC* and CCNE1 alterations were present in one patient with an exceptional prolonged response, highlighting the influence that DDR alterations may have on response to treatment. 

A recent randomised phase II study assessed the combination of AZD1775 and gemcitabine, versus gemcitabine monotherapy, in platinum resistant HGSOC [105]. Impressively, a survival benefit was observed, with median Overall Survival 11.5 months with combination treatment, compared to 7.2 months in the gemcitabine arm (Hazard Ratio 0.56, 95% Confidence Interval 0.34–0.92; *p* = 0.022). A partial response was observed in 13 (21%) of the patients in this study. Given the survival benefit observed, this approach warrants further investigation in a phase III trial. 

### 4.4. BET Inhibitors 

Bromodomains are small protein domains that recognise and bind to acetylated histone tails, modify chromatin structure, and lead to upregulation of target genes to drive oncogenesis [106]. BET inhibitors reversibly bind to bromodomains of BET proteins, blocking interaction with acetylated histones and transcription factors. Inhibition of BET interferes with *BRCA1* and *RAD51* expression [107]. BET inhibition has been demonstrated to induce HRD in ovarian cancer cell lines, by decreasing transcription of BRCA1 and RAD51, depleting the DNA double stand break resection protein CtIP (C-terminal binding protein (CtBP) interacting protein) and downregulating the G2-M cell-cycle checkpoint regulator WEE1 and the DNA-damage response factor TOPBP1 [107,108,109,110]. BET and PARP inhibition has demonstrated a synergistic effect on reducing xenograft tumour growth in HR-proficient ovarian cancer mouse models [107,108]. These effects have been shown to be independent of *BRCA1/2*, *TP53*, *RAS*, and *BRAF* mutation status. Combining PARP and BET inhibitors may thus help overcome not only primary resistance but also the development of secondary resistance.

### 4.5. Anti-Angiogenic Therapies 

Preclinical studies have demonstrated that anti-angiogenic therapy can induce a hypoxic tumour microenvironment, which is associated with downregulation of HR genes [111]. This is the mechanistic rationale for investigating the combination of anti-angiogenic therapy with PARPi, to enhance their synthetically lethal effects. 

A single arm phase II study [112] assessing the combination of olaparib and cediranib demonstrated an ORR of 20% in a platinum resistant OC cohort. The observed benefit appeared to be higher in patients with germline *BRC*Am. The Evolve study [113] was a proof of concept clinical-translational phase II trial of cediranib-olaparib in ovarian cancer, including patients who had acquired resistance to prior PARPi therapy, enrolled into platinum sensitive (*n* = 10), platinum resistant (*n* = 10) and exploratory (*n* = 10) cohorts. Two partial responses were observed amongst a cohort of 10 patients with platinum-resistant OC. The 16-week PFS was 54.5% (31.8–93.6) in PS, 50% (26.9–92.9) in PR and 36% (15.6–82.8) in PE, respectively. OS at one year was 81.8% (61.9–100) in PS, 64.8% (39.3–100) in PR and 39.1% (14.7–100) in PE. Correlative analyses identified mechanisms of PARPi resistance in ~77% of evaluable patients with matched pre-post PARP inhibitor progression biopsies, such as reversion mutations in *BRCA1/2* and other HR genes, MDR1 upregulation, CCNE amplification and RIG-I like receptor downregulation [113]. The anti-tumour activity of this combination will be assessed further in the OVC2 trial (NCT02502266), a randomised phase III study evaluating Olaparib+Cediranib vs. chemotherapy in Platinum Resistant OC. Importantly, this study demonstrates the feasibility of integrating translational objectives into clinical trial protocol design. This is critical, as further translational studies to exploring mechanisms of treatment resistance will be crucial to developing more effective therapeutic strategies. 

### 4.6. G-Quadruplex Stabilisation 

G-quadruplex (G4) structures can potentially form at over 700,000 sequences in the human genome, including telomeres, rDNA, the immunoglobulin heavy chain switch regions, minisatellite and microsatellite repeats [114,115,116]. G4 structures increase the tendency for DNA damage to occur, by impeding DNA polymerase and DNA damage repair processes. Chromosome breaks induced by G4 structures will activate diverse repair pathways, such as HR, TLS, NHEJ pathways and pol θ-mediated alternative end-joining pathways [117,118,119,120]. CX-5461, is a novel, synthetically derived small molecule, which selectively kills HR deficient cancer cells, through stabilising G4 structures and inducing replication-dependent DNA damage. Phase 1 studies of CX-5461 in solid tumours have been completed [121], with further investigation planned in an ovarian cancer cohort with acquired resistance to treatment from prior PARPi and/or platinum exposure.

## 5. Overlap between Acquired Platinum and PARPi Resistance Mechanisms

**Figure 2:**
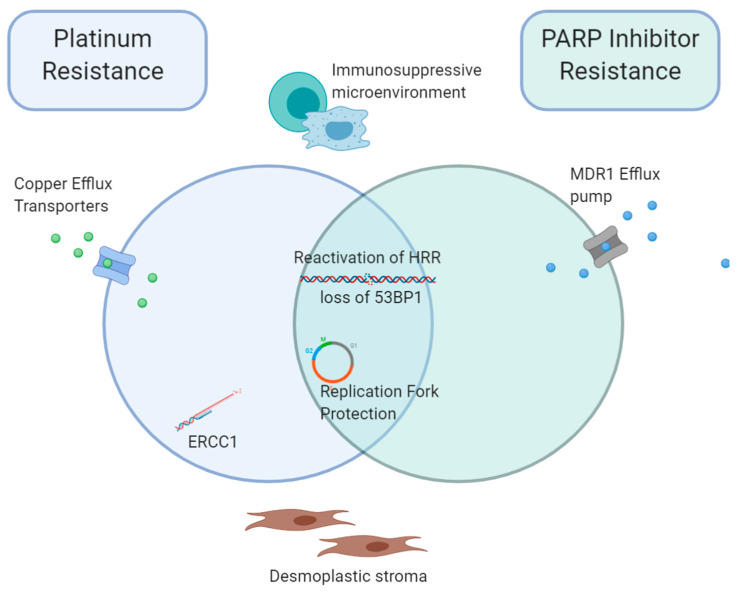
Overlapping mechanisms of resistance to Platinum and PARP-inhibitor treatment. Platinum and PARPi demonstrate distinct and overlapping mechanisms of acquired treatment resistance. Whilst both demonstrate reactivation of HRR and loss of 53BP1, and replication fork protection promoting progression through the cell cycle, platinum resistance is additionally associated with defects in the NER pathway, including increased ERCC1 activity. Reduced intracellular drug accumulation is achieved via copper transporters for platinum, and MDR1 drug efflux pump for PARPi. In both instances, an immunosuppressive microenvironment and desmoplastic stroma likely contribute to tumour progression. Abbreviations; HRR, homologous recombination repair, MDR1, Multidrug Resistance Protein 1, ERCC1, DNA excision repair protein ERCC-1, PARPi, PARP inhibitor.

Clearly, there is significant overlap between mechanisms of resistance to platinum chemotherapy, and PARPi, with DDR alterations playing a key role. However, it must be noted that patients who progress on PARPi maintenance often retain sensitivity to platinum chemotherapy. Acquired resistance to these classes of agents clearly does not develop entirely in parallel (Figure 2). It is not yet clear whether patients who progress on PARPi, then respond to platinum chemotherapy, may retain some sensitivity to PARPi and benefit from second maintenance therapy with PARPi. This question is currently being explored in the OReO study (NCT03106987; ENGOT-ov38/OReO), a Phase IIIb study of olaparib maintenance retreatment in patients with epithelial ovarian cancer previously treated with a PARPi and responding to repeat platinum chemotherapy. It will also be addressed in the olaparib monotherapy arm of the DUETTE study (NCT04239014). With the success of PARPi as first and second-line maintenance treatment, a new patient population with platinum sensitive relapsed OC is emerging, for whom no standard of care exists. Hence, the results of these studies are eagerly awaited, for their capacity to define a new standard of care in this patient population of unmet needs. 

## 6. Biomarkers of Resistance to PARPi and Platinum

Predicting platinum or PARPi resistance in individual patients remains challenging, as resistance mechanisms are multifactorial and evolve over time. As discussed earlier, whilst current HRD assays have their limitations, HRD is a powerful biomarker for predicting the initial response to both platinum chemotherapy and PARPi [26]. At time of relapse, the Therapy Free Interval (TFI), defined as the time between last treatment and documented relapse, remains the most widely used clinical predictor of likely response [122]. Recently, however, the biological relevance of using a six-month TFI to define platinum resistance has been challenged, and much effort has been put into defining and validating molecular biomarkers of treatment resistance [123]. The detection of somatic mutations linked to acquired resistance mechanisms (such as *BRCA* reversion mutation) in ctDNA is a promising non-invasive biomarker, which warrants investigation in future prospective studies. 

## 7. Conclusions

Platinum chemotherapy remains the cornerstone of systemic therapy for OC, with PARPi playing a key role as maintenance treatment. Resistance to these treatments has important prognostic implications. Whilst research has improved our knowledge thus far, our global mechanistic understanding is still limited. In particular, the complex interactions, compensatory changes, and relative contributions between DDR pathways and the tumour microenvironment remain to be fully described. The incorporation of translational objectives into clinical trial protocol design will enable further elucidation of these important mechanistic questions, in order to improve patient stratification, and develop therapeutic approaches that effectively target molecular vulnerabilities in treatment-resistant OC. 

## Figures and Tables

**Table 1:** Examples of current active trials that target DNA Damage Repair pathways to overcome resistance to PARPi and Platinum.

Study Name/NCT	Target	Study Treatment	Study Population	Study Phase
DUETTE NCT04239014	ATR/PARP	Ceralasertib (AZD6738) + Olaparib, or Olaparib monotherapy, or Placebo	Relapsed platinum-sensitive OC, who have acquired resistance from prior PARPi treatment	II, RCT
NCT02723864	ATR/PARP	VX-970 + Veliparib and Cisplatin	Advanced refractory solid tumours	I
NCT02901899	DNMT/PD-1	Guadecitabine + pembrolizumab	Recurrent Platinum Resistant OC	II, open-label
NCT03924245	HDAC/PARP	Entinostat + Olaparib	Recurrent platinum refractory and resistant EOC	I/II
NCT02915523	PD-L1/HDAC	Avelumab ± Entinostat	Advanced OC Which Has Progressed or Recurred After First-line Platinum-based Chemotherapy and at Least Two Subsequent Lines of Treatment	Ib/II, RCT
NCT02797977	CHK1	SRA737 + gemcitabine + cisplatin, or gemcitabine monotherapy	Advanced solid tumours, including HGSOC which is *BRCA1* and *BRCA2* wild type.	I/II, non-randomised
NCT03057145	CHK1/PARP	(Prexasertib) LY2606368 + Olaparib	Advanced solid tumours	I
NCT03579316	WEE-1/PARP	Adavosertib (AZD1775) + Olaparib, or adavosertib monotherapy	Recurrent OC with progression on prior PARPi therapy	II, RCT
NCT02502266	Angiogenesis/PARP	Cediranib + Olaparib, or chemotherapy	Platinum resistant or Refractory OC	III, RCT

Abbreviations; ATR, ataxia telangiectasia and Rad3-related protein; PARP, Poly (ADP-ribose) polymerase; DNMT, DNA methyltransferase, PD-1, Programmed cell death protein 1; PD-L1, Programmed death-ligand 1; HDAC, Histone deacetylases; CHK1, Checkpoint kinase 1; RCT, Randomised Control Trial.

**Table 2:** Contribution of DNA Damage Repair pathway alterations to Platinum and PARPi resistance.

DNA Damage Repair Pathway	Key Pathway Functions	Key Genes	Effect of Pathway Alterations on Therapeutic Resistance
Homologous Recombination (HR)	Repair of DSBs or stalled replication forks during S and G2 phases of cell cycle	*BRCA1*, *BRCA2*, *RAD51*, *HSP90*	Reactivation of HR pathway enables repair of DSBs and resolves replisome blocks, promoting cancer cell progression through the cell cycle despite the presence of cytotoxic DNA damage.
Non-Homologous End Joining (NHEJ)	Repair of DSBs during interphase	*53BP1*	Loss of *53BP1* re-wires NHEJ pathway, reactivating HR independent of BRCA1
Base Excision Repair (BER)	Repair of SSBs and DNA base lesions	*PARP-1*, *XRCC1*, *Pol β*	Functional BER pathway leads to loss of synthetic lethality and PARPi resistance
Nucleotide Excision Repair (NER)	Removes “bulky lesions” which distort the DNA double helix, including intra-strand crosslinks formed by platinum adducts.	*ERCC1*, *XPF*	Upregulation of *ERCC1* and *XPF* potentially restores NER function. NER pathway alteration potentially confers sensitivity to platinum, and not PARPi.
Fanconi Anemia (FA)	Removes intra-strand DNA crosslinks, coordinates DNA replication by fine-tuning mitotic checkpoints and replication fork stabilisation	*FANCC*, *FANCD2*, *FANCA*	Mutations in FA pathway genes may have a similar effect to *BRCA1* and *BRCA2* mutation, in promoting progression of cancer cell through the cell cycle, even in setting of DNA damage and replication stress
Mismatch Repair (MMR) Deficiency	Recognise, excise and resynthesise mismatched or unmatched DNA base pairs or insertion-deletion loops.	*MLH1*, *MSH2*	MMR deficiency results in microsatellite instability, interfering with detection of cytotoxic DNA damage, allowing cancer cells to proliferate despite DNA damage.

DSBs; Double Strand DNA breaks, SSBs; Single Strand DNA breaks, PARPi; PARP inhibitor.

**Table 3:** Mechanisms of reactivation of Homologous Recombination repair.

Resistance Mechanism	Function
*BRCA* (or *HR* gene) reversion mutation	Restores open reading frame of gene, resulting in functional protein expression
Loss of *BRCA1* promoter methylation	Restores BRCA1 function
Upregulated HSP90	Promotes BRCA-independent RAD51 loading onto damaged DNA
BRCA1 C-terminal domain mutation	Upregulation of BRCA1, in absence of BRCA1 reversion mutation
Loss 53BP1	Recruits Shieldin complex to inhibit DNA resection, initiating HR in a BRCA-independent manner.

Abbreviations; HR, Homologous Recombination; HSP90, Heat Shock Protein 90.
